# The Uniform Pattern of Growth and Skeletal Maturation during the Human Adolescent Growth Spurt

**DOI:** 10.1038/s41598-017-16996-w

**Published:** 2017-12-01

**Authors:** James O. Sanders, Xing Qiu, Xiang Lu, Dana L. Duren, Raymond W. Liu, Debbie Dang, Mariano E. Menendez, Sarah D. Hans, David R. Weber, Daniel R. Cooperman

**Affiliations:** 1 Department of Orthopaedics and Rehabilitation, University of Rochester, Rochester, New York USA; 2 Department of Biostatistics and Computational Biology, University of Rochester, Rochester, New York USA; 3 Department of Orthopaedic Surgery, University of Missouri, Columbia, USA; 4 Department of Orthopaedic Surgery, Case Western Reserve University, Cleveland, Ohio USA; 5 Department of Orthopaedic Surgery, University of California San Francisco, California, USA; 6 Department of Orthopedics, Tufts University School of Medicine, Boston, Massachusetts USA; 7 Colon Rectal Specialists, Rochester Hills, Michigan USA; 8 Department of Pediatrics, University of Rochester, Rochester, New York USA; 9 Department of Orthopaedics and Rehabilitation, Yale University, New Haven, Connecticut USA

## Abstract

Humans are one of the few species undergoing an adolescent growth spurt. Because children enter the spurt at different ages making age a poor maturity measure, longitudinal studies are necessary to identify the growth patterns and identify commonalities in adolescent growth. The standard maturity determinant, peak height velocity (PHV) timing, is difficult to estimate in individuals due to diurnal, postural, and measurement variation. Using prospective longitudinal populations of healthy children from two North American populations, we compared the timing of the adolescent growth spurt’s peak height velocity to normalized heights and hand skeletal maturity radiographs. We found that in healthy children, the adolescent growth spurt is standardized at 90% of final height with similar patterns for children of both sexes beginning at the initiation of the growth spurt. Once children enter the growth spurt, their growth pattern is consistent between children with peak growth at 90% of final height and skeletal maturity closely reflecting growth remaining. This ability to use 90% of final height as easily identified important maturity standard with its close relationship to skeletal maturity represents a significant advance allowing accurate prediction of future growth for individual children and accurate maturity comparisons for future studies of children’s growth.

## Introduction

Accurately predicting children’s future growth, while important for many specialties, remains challenging. Our present study’s hypotheses are that timing relative to the growth spurt and skeletal maturity are highly correlated with growth remaining and can accurately predict future growth. Childhood growth is characterized by three major phases: a rapid decelerating infantile growth phase lasting until approximately age 3; a longer childhood phase with a steady height increase; and the adolescent growth spurt marked by an initial period of rapidly accelerating height velocity reaching a maximum rate at the peak growth age (PGA) followed by deceleration until final mature height. Children tend to remain in a constant percentile relative to same sex peers during the childhood phase, but cross percentiles by entering the adolescent growth spurt at differing times. This variation in timing limits the utility of cross sectional studies to predict growth. Because humans are one of few species undergoing an adolescent growth spurt, animal studies also have limited utility. These constraints make longitudinal human studies essential to understanding human growth. Prior studies have not identified markers of growth sufficiently reliable for clinically accurate growth predictions. Predicting the timing or magnitude of future growth requires a model derived from a robust longitudinally evaluated population of children with closely spaced intervals of height measurement and other predictive details. Several mathematical models have been proposed to model growth, but are better at description than prediction^1^.

Our understanding of human growth patterns derives from studies following children longitudinally with serial anthropometrics^2–12^. These studies demonstrate that the timing of the adolescent growth spurt is non-uniform, boys undergo their growth spurt about two years later than girls, children change their age matched percentiles during the growth spurt, and the pattern of growth during the growth spurt is similar between children. The change in percentiles as a child ages can be very impressive as some children are growing rapidly at one time while their peers are not. Our understanding of radiographic skeletal maturation similarly derives from those longitudinal studies which obtained serial radiographs concurrent with anthropological measurements. Lacking the necessary computational power and statistical techniques of analyzing complex longitudinal data now available, most previous investigations analyzed the longitudinal data using cross sectional techniques of chronological age percentiles, averages, and standard deviations, which unfortunately blunt the individual growth curves and hide accelerations and curve shape similarities.

Our present study’s hypotheses are that (1) timing relative to the growth spurt, specifically the PGA, is highly correlated with growth remaining, and (2) that skeletal maturity using previously described, reliable methods from hand radiographs accurately predicts growth remaining. We tested these hypotheses via longitudinal analyses of two prospective studies, comparing the PGA to percentage of final height and skeletal maturation and identify a uniform pattern of growth during the human adolescent growth spurt closely related to skeletal maturity to providing future growth predictions by percentage of adult height achieved.

## Methods

### Study populations

The Brush Foundation Study of Child Growth and Development is the largest and most complete longitudinally collected collection of combined anthropometrics and skeletal radiographs. The prospective study started in 1931 enrolling healthy children from 3 months to 14 years of age during each successive year until 1942. Subjects had measurements and radiographs every 3 months until 1 year of age, every 6 months until age 5 and annually thereafter. The study contains records of 4483 children with follow-up ranging from two to twelve years^6^. This study is most well-known for the Greulich and Pyle^13^ hand and wrist skeletal maturity atlas.

The Berkeley Guidance Studies of the Institute of Human Development^14^, best known for Bayley’s work on mental development^15,16^, enrolled every third child born in Berkeley in 1928–9 and followed them to growth completion with serial anthropometrics and skeletal maturity radiographs; however, the radiographs no longer exist.

### Subject selection

From the Brush study, height and height velocity plots were created for each subject potentially undergoing their adolescent growth spurt during data collection. We identified those who both exhibited the adolescent growth spurt and completed growth, defined as less than 1 cm/year between the final annual visits. All Berkeley subjects were included having undergoing both their growth spurt and growth completion.

### Institutional review board

All methods were carried out in accordance with relevant guidelines and regulations and was approved by the University Hospitals Case Medical Center Institutional Review Board (IRB). The subjects for the Brush study were enrolled by their parents in the 1920s and 30s, and knowingly participated in this longitudinal study. The Berkeley data was obtained from prior published information with children similarly enrolled.

### Determination of PHV

The velocity of maximum growth during the mid-growth spurt is termed the peak height velocity (PHV). We termed the age of PHV the “peak growth age (PGA)”. To determine the PGA, we first fit growth curves using cubic splines to the discrete data and denoted the fitted growth curves by ${\hat{h}}_{i}(t)$. The derivatives of fitted growth curves, $\hat{h}{\text{'}}_{i}(t)$, were used as estimates of the height velocity functions. Peak height velocity (PHV) of the *i*th subject is estimated by taking the maximum of $\hat{h}{\text{'}}_{i}(t)$ over the time interval, $[{\rm{\max }}({t}_{i1},{T}_{{\rm{\min }}}),{t}_{i{J}_{i}}]$, where ${t}_{i1}\,$is the first and smallest time point; ${J}_{i}$ is the number of longitudinal observations of the *i*th subject and ${t}_{i{J}_{i}}$ is the last and largest time point. ${T}_{{\rm{\min }}}$ is a pre-specified minimum age for PGA (9.0 years for girls and 10.0 years for boys). The corresponding time point is used as the estimate of PHV. If PHV occurs at the two boundary points (either ${t}_{i1}$ or ${t}_{i{J}_{i}}$), extrapolation is used to determine the PHV. If the extrapolated height velocity does not attain a maximum, which can happen if it is monotonically increasing (decreasing) outside of the right (left) boundary points, a missing value is reported. With the estimated PGA, we are able to classify maturity as timing pre- or post-PGA. The timing before and after PGA in years^9,10,17^ was compared to the percentage of final height. The reciprocal of the percentage final height is the individual specific height multiplier^18,19^.

### Comparison of skeletal maturity to growth completion

Radiographic skeletal maturity was assessed using three separate reliable^20–24^ techniques of the hand and wrist: the Greulich and Pyle (GP) method^13^, the Fels^24^ method, and the Sanders simplified method^23^ which was derived from the Tanner-Whitehouse 3 method^25^, using separate readers for each method. The relationships between skeletal maturity and the growth parameters were represented as penalized cubic-splines based on functional data analysis techniques^26,27^. Specifically, we chose integrated squared second derivative of the smoothed curves as the roughness penalty. The smoothing parameter is determined by the generalized cross-validation (GCV) criterion.

### Data availability statement

The datasets generated during and/or analyzed during the current study are available from the corresponding author on reasonable request.

## Results

For the Brush study, 54 subjects completed growth at study terminus (35 f, 19 m). The age of girls at their first visit ranged from 2 to 10 years and boys from 7.5 to 11. The Berkeley study had 135 subjects (69 f, 66 m) and all were followed from infancy. The final adult heights and peak growth ages are shown in Table 1. Across all ages compared to the most recent NHANES data^28^, the Brush series average and median percentiles were respectively 56.5ptl and 60.5ptl for girls and 60.9ptl and 62.8ptl for boys. For the Berkeley study, the corresponding values were 63.1ptl and 68.6ptl for girls 61.6ptl and 65.0ptl for boys.

**Table 1:** Population Data including adult height, age at peak height velocity or peak growth age (PGA), Age at 90% of adult height, and the % adult height attained at PGA.

	Brush		Berkeley	
	Girls	Boys	Girls	Boys
Adult Heights	151–175 (mean 163.4) cm	169–183.9 (mean 177) cm	153.6–183.2 (mean 166.5) cm	161.8–195.1 (mean 179.6) cm
PGAs (spline)	9.7–13.4 years (mean 11.3 ± SD 0.86 yr)	11.7–14.3 years (mean 13.0 ± SD 0.65 yr)	9.0–14.4 years (mean 11.5 ± SD 1.1 yr)	9.0–15.0 years (mean 13.4 ± SD 1.4 yr)
Age at 90% adult height	9.9–12.6 years (mean 11.2 ± SD 0.68 yr)	11.9–14.1 years (mean 13.0 ± SD 0.6 yr)	9.6–13.2 years (mean 11.4 ± SD 0.8 yr)	11.6–15.8 years (mean 13.5 ± SD 0.9 yr)
% adult height at PGA	83.4% − 96.5% (mean 90.0% ± SD 2.5%)	85.6% − 93.8% (mean 90.0% ± SD 2.1%)	81.2% − 94.8% (mean 90.5% ± SD 2.4%)	75.3% − 94.8% (mean 90.2% ± SD 4.0%)

### PGA relative to 90% final height

**Figure 1:**
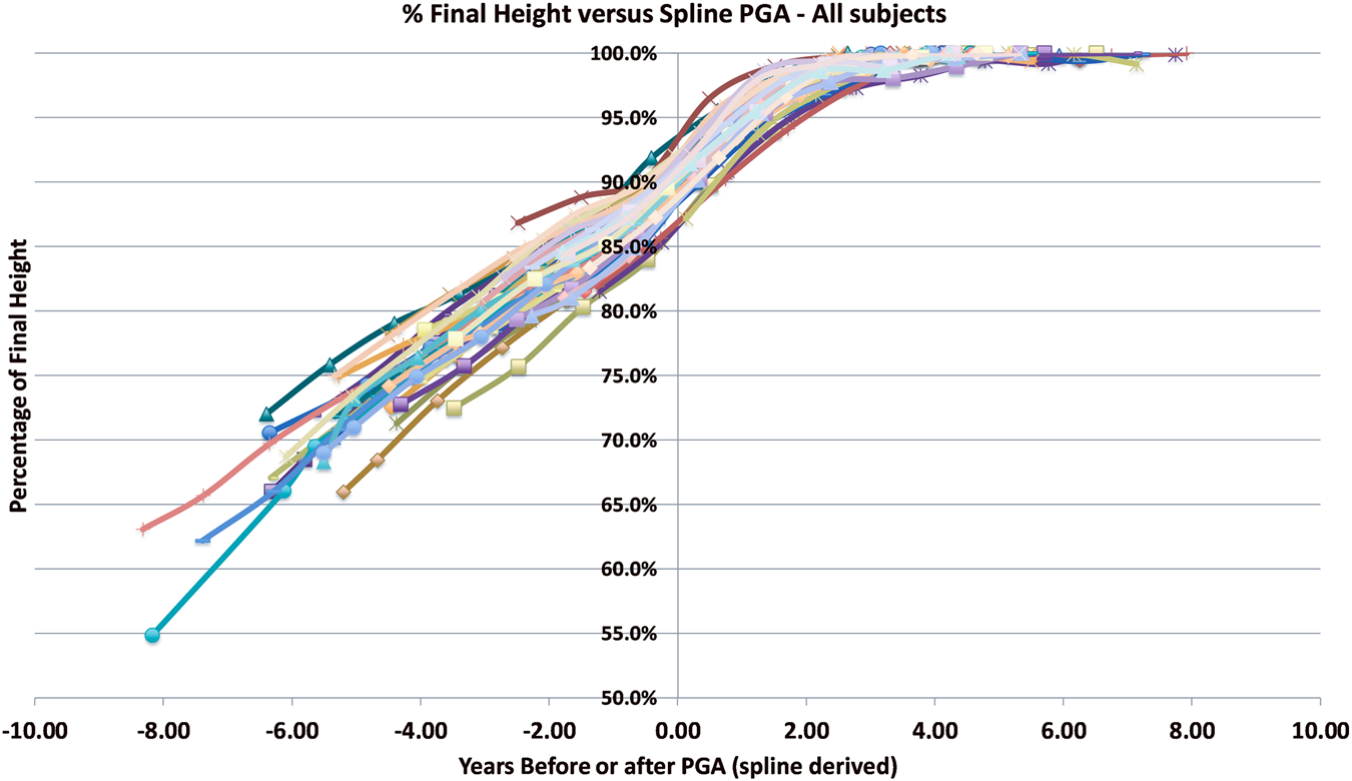
Graph of Brush Series Using Chronological Age. Graph of Brush Series showing the pattern similarity between all the subjects comparing timing before and after their peak growth age (PGA) derived from spline curve 1st derivatives to the percentage of final height attained.

**Figure 2:**
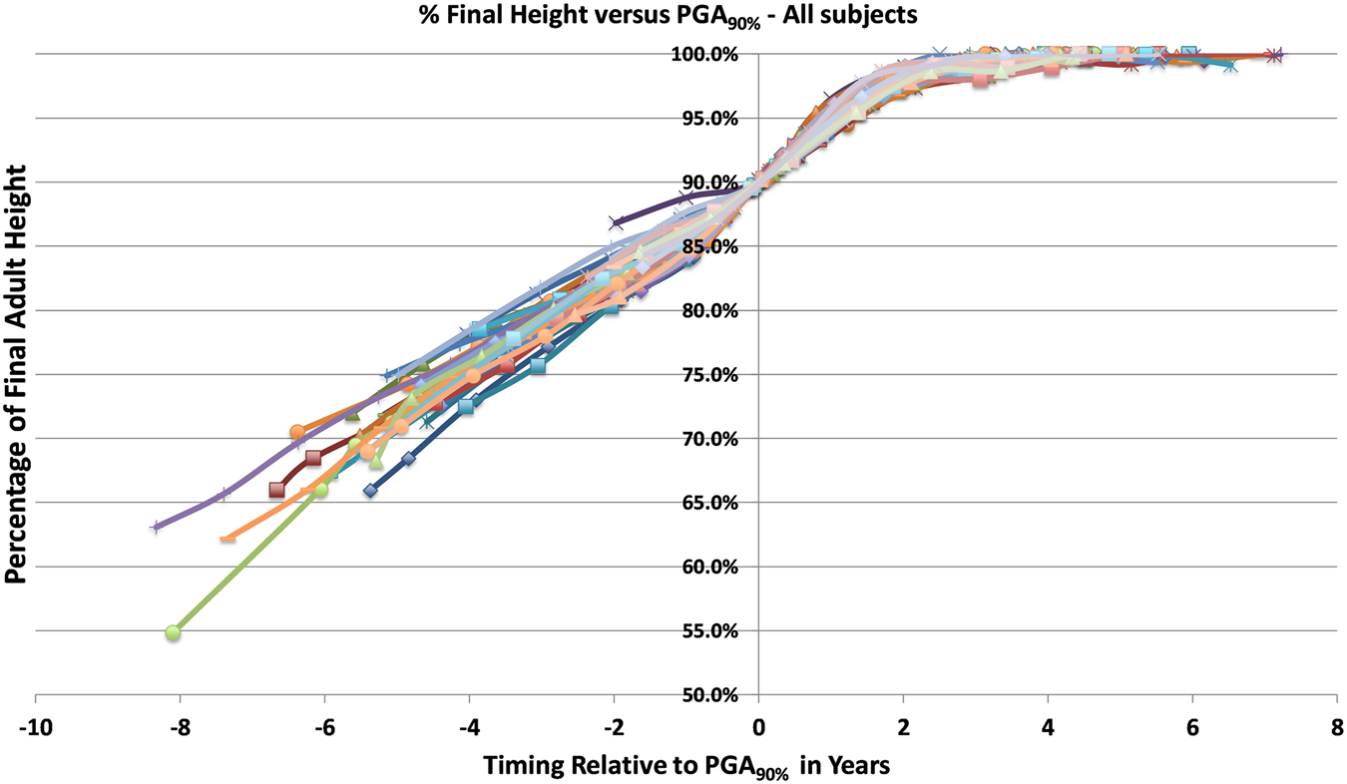
Graph of Brush Series % Adult Height to PGA_90%_. Graph of Brush Series showing growth curves of all the subjects comparing timing before and after attainment of 90th percentage of adult height attained (PGA_90%_).

In our assessment of the Brush data, the shape of the curves for all subjects was quite similar (Fig. 1). Indeed, the curves appeared phase shifted about the mean PGA of 90% final height, which was the mean PGA for both boys and girls. Performing the phase shift such that 90% of final height occurred at the same point demonstrated that the growth curves were very similar for all subjects, both boys and girls and provided very tight fitting of overall height growth between all subjects of both sexes (Fig. 2). The 90% final growth timing we termed the PGA_90%_. With this phase shift, the percentage growth remaining curves begin to coalesce at about 85% of final growth and remained very consistent from 90% until final maturity. The mean difference between the spline derived PGA and the age at 90% final height was 0.03 years (std. dev. 0.32 years), or essentially equivalent since the measurements were annual.

**Figure 3:**
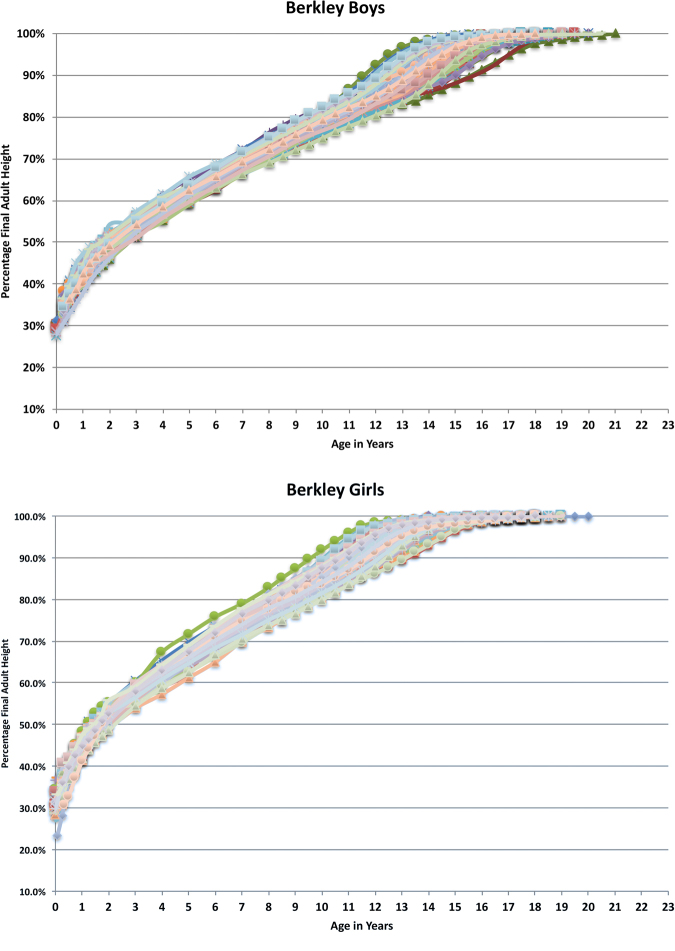
(**A**) Percentage of Final Height of Berkeley Boys by Chronological Age. Percentage of final height of Berkeley boys showing the wide distribution of remaining heights by chronological age about the growth spurt. (**B**) Percentage of Final Height of Berkeley Girls by Chronological Age. Percentage of final height of Berkeley girls showing the wide distribution of remaining heights by chronological age about the growth spurt.

**Figure 4:**
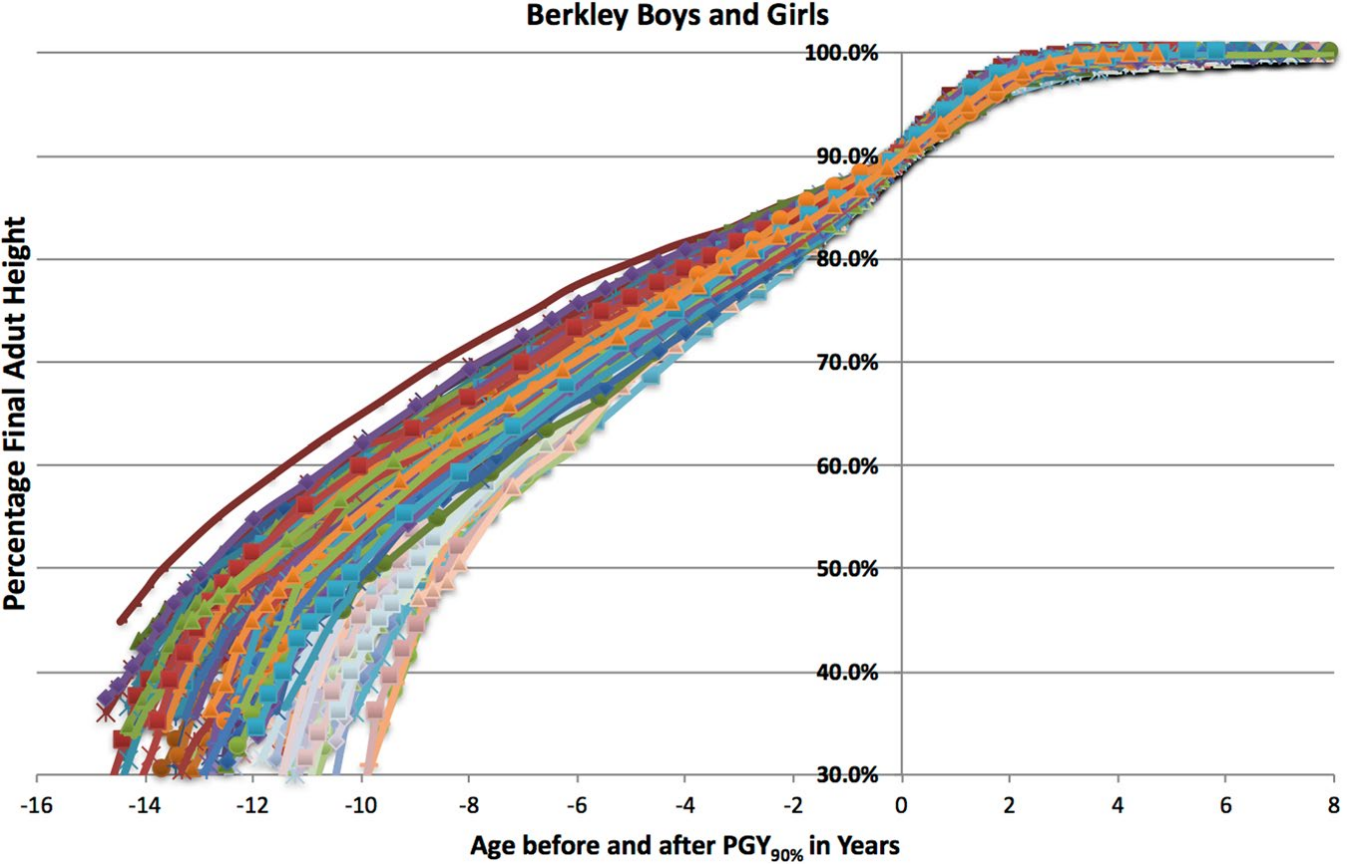
Percentage of Adult Height of Berkeley Boys and Girls by Age Compared to PGA_90%_. The percentage of adult height for both boys and girls compared to PHV timing adjusted to 90% final height (PGA_90%_) showing similar pattern for both boys and girls.

We performed the same assessment for the Berkeley subjects and obtained identical growth patterns. The percentage of final height for the Berkeley children compared to chronological age is shown in Fig. 3a (for boys) and 3B (for girls). The percentage of final height for both boys and girls compared to PHV timing adjusted to 90% final height which we designated as PGA_90%_ is shown in Fig. 4. The curves are clearly very similar for both boys and girls. The comparative data of the Berkeley to Brush subjects for both age at 90% adult height and percentage final adult height at PGA are shown in Table 1.

Comparing the Berkeley and Brush series showed no statistical differences between mean PGA and mean age at 90% height for both sex groups. No significant difference in growth trends were detected at point-wise confidence level $\alpha =0.05$ based on a functional *t*-test^27^ that investigates the statistical significance of mean differences between two sets of height growth curves.

### Skeletal maturation

**Figure 5:**
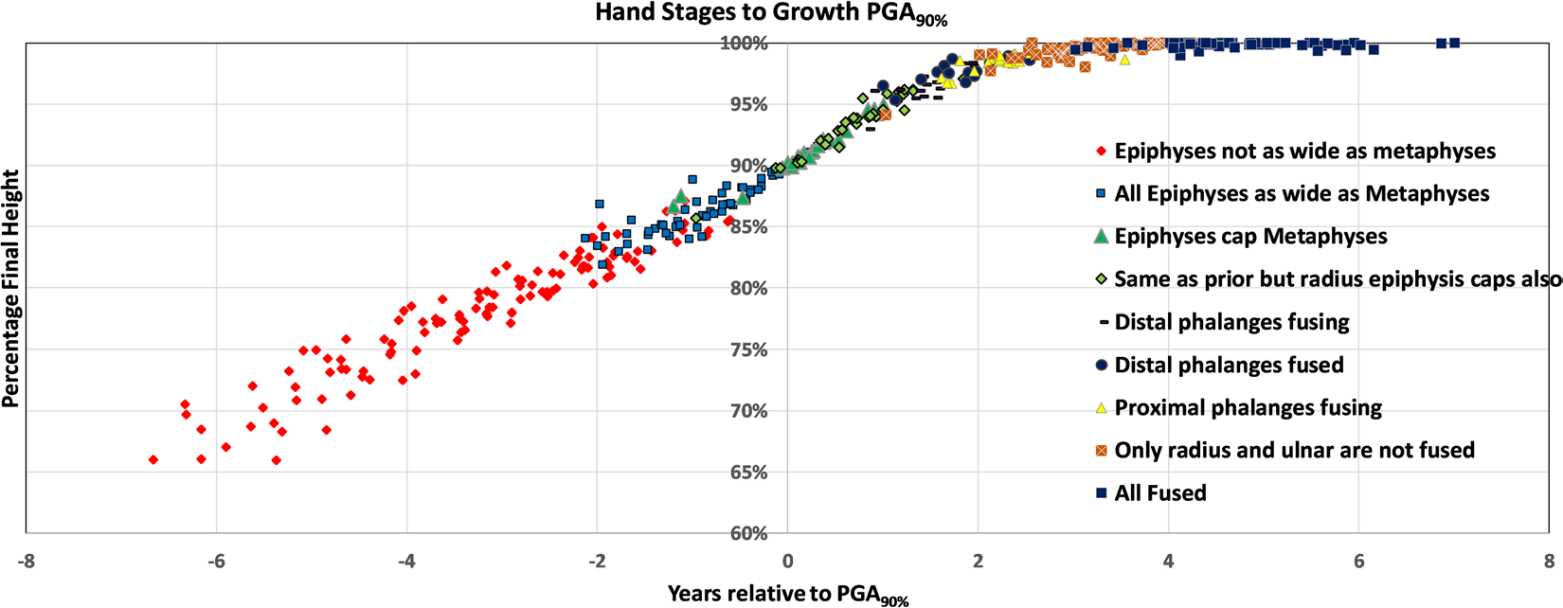
Hand Stages compared to PGA_90%_. Stages as described by Sanders demonstrating the morphology of the hand growth centers compared to 90% final height (PGA_90%_) and growth remaining.

Table 2 compares the results of the three skeletal maturity measurements. The residuals demonstrate very small variability. In children undergoing the adolescent growth spurt (Sanders $\ge$2) whether evaluating for timing relative to the PGA_90%_ (all the measures have a SD of ~0.5 year) or percentage final height (all SD <2%). The residuals are slightly wider when including preadolescents (Sanders score 1 subjects), as shown in Table 2. Together, these results suggest that skeletal maturity convergences as children enter their growth spurts. Among the three maturity measures, the Fels method is the most reflective of the final height and relative age to PGA_90%_ although the Sanders score performs very well during the adolescent growth spurt for which it was designed and exhibits a floor effect prior to the growth spurt. Sanders stages are directly reflective of the hand epiphyseal morphological appearance allowing sex independent radiographic morphology to be directly related to both timing relative to PGA_90%_ and percentage of final height which is similar for all the children (see Fig. 5). When the epiphyses are as wide as the metaphyses (Sanders stage 2, Tanner-Whitehouse stage F) the child has entered their adolescent growth spurt at 85% of adult height, 2 years before the peak growth. The peak is reached at Sanders stage 3 when all the digits are capping (Tanner-Whitehouse stage G), at 90% of final height. The growth spurt slows markedly at 1.5 years after the peak at 96% of final height when the distal phalanges begin to fuse (Sanders stage 4 and 5, Tanner-Whitehouse stage H), and then asymptotically approaches adult height at 3.5 years following the peak growth. Growth is nearly complete when only the distal radius and ulnar physes are open. The relationship between skeletal maturity and percentage final height, timing relative to PGA_90%_ and the multipliers for the Fels, Greulich and Pyle, and Sanders methods from univariate analysis are included in the supplemental materials.

**Table 2:** Mean residual standard deviations for skeletal maturity scales compare to PGA90% and percentage of adult height. For the ‘preadolescents excluded’, the data was only evaluated once the child entered their adolescent growth spurt (Sanders score $\ge$2).

Sex	Timing to PGA_90%_ (years)				% Adult Height (%)			
	All Subjects		Preadolescents Excluded		All Subjects		Preadolescents Excluded	
	Males	Females	Males	Females	Males	Females	Males	Females
Sanders	0.927	0.901	0.567	0.626	2.693	3.006	1.499	1.488
GP	0.689	0.672	0.583	0.677	2.029	1.935	1.771	1.752
Fels	0.671	0.605	0.587	0.581	1.726	1.883	1.372	1.490

## Discussion

Standard height prediction methods in children take into consideration current height and either chronological or skeletal maturity percentiles with the assumption that the child will remain a similar percentile through growth. This is not the case when children go through their growth spurt at differing times. The development of statistical techniques for the assessment of longitudinal growth data provide the ability to analyze existing data in ways previously unavailable to prior investigators. Using data and radiographs from two prior studies, we identified the coincidence of PHV timing and completion of 90% final height attainment in both boys and girls. When the children’s heights are normalized to final height and standardized by timing relative to PGA_90%_, the growth patterns of both boys and girls become identical during the adolescent growth spurt beginning at about 85% of final height. Similarly, skeletal maturation of the hand is identical for both boys and girls from the initiation of the adolescent growth spurt at 85% final to growth completion.

Our findings were anticipated by these earlier investigators who lacked the necessary statistical and computational tools for longitudinal data analysis which developed subsequent to their publications. In 1937, Shuttleworth^29^ graphically phase shifted height velocity growth curves and noted their overall shape and duration similarity. Bayley, in 1943^30,31^ and 1952^32^ working with the Berkeley study, related growth to percentage final height rather than cross sectional percentiles. Similar to our results, she noted that when the sexes are compared using skeletal maturity to final percentage height, boys and girls are nearly identical^30^:

When percentage of mature height is classified according to chronological age there is considerable spread of the percentage values except close to the point of maturity. But when skeletal age is made the basis for groupings, the (standard deviations) shrink markedly, showing that relative size is much more closely related to skeletal maturity than to chronological age.

Interpolating Bayley’s results identifies the 90% final height at chronological age 11.4 years for girls and 13.7 years for boys, nearly identical to our findings. The corresponding Greulich and Pyle skeletal ages were 11.0 for average girls and just under 13.5 years for boys, also corresponding to our finding of maturity using the Brush data. Buckler’s study performed 40 years after the Brush study, found that PGA occurred at 90⋅9% final height in boys and 90⋅7% in girls, again, nearly identical to our results. While other investigators have examined skeletal maturity relative to the PGA^33–39^, they did not directly compare this with percentage adult height attainment.

All modern skeletal maturity assessment scales were developed by identifying orderly, reliably occurring radiological morphologic changes. The three hand maturity scales in this study were selected for different properties. The Greulich and Pyle method is the most commonly used technique in the USA and assigns a “skeletal age” based upon the modal radiographic appearance from the Brush collection of same sex children. The Fels method is a detailed scoring system based upon 98 separate indicators which receive a specific score based upon chronological age and sex. These scores are then placed into an empirical maximum-likelihood algorithm which generates a skeletal age. The Sanders stages only applies to adolescent growth, and performed very well during this phase. The hand stages are nearly identical between boys and girls at the same growth spurt stage. Our results imply that using skeletal maturity appearance itself rather than separate male and female skeletal ages should allow more reliable study of the human growth spurt. The Fels system, which is the most complex, relates very closely to growth remaining and can likely become more functional with computerized radiographic reading. When detailed assessment is justified, the Fels method provides the best indicator for all subjects. The Greulich and Pyle also performs well, and the Sanders staging performs very well once the growth spurt begins. The coalescence of skeletal maturity as a child enters the growth spurt has been noted by other investigators^33^. From our data, it makes sense to develop future skeletal maturity scales whose distinguishable phases directly reflect growth remaining rather than simple sequential occurrence. Skeletal maturation of the hand follows an orderly pattern making it no surprise that the appearance of an individual adolescent’s radiographs corresponds to percentage growth remaining once a child reaches their adolescent growth spurt. It can also provide further tools for comparative anthropological and biomedical inquiries.

The percentage of growth remaining is a clinically and anthropologically useful means of determining current maturity and predicting future growth. The multiplier is the reciprocal of the percentage growth remaining which is further justified in humans by our work. During the adolescent growth spurt, the correlation of growth remaining is excellent using skeletal maturity and much poorer with chronological age. Prior to the growth spurt, the growth curves have not coalesced into the shape occurring during the growth spurt. Prior work studying the lower extremities demonstrated excellent prediction of extremity length using a skeletal maturity derived multiplier during the adolescent growth spurt while predictions before to the spurt were not improved with skeletal over chronological maturity^40^. The multiplier method has the advantage of eliminating the need to work with percentiles. A taller individual will grow proportionally more than a shorter individual with the same multiplier. Expressing this in the more commonly used centiles, a child at the 10^th^ percentile will grow the same relative amount during the growth spurt as one at the 90^th^ percentile though their absolute gains are different. Exactly why this occurs is uncertain, but because nearly all longitudinal growth is physeal from either the long bones or the spine, the changes resulting in the final 15% of growth must occur through the physis whether through increased proliferation or longer hypertrophic cartilage columns. This process is likely intrinsic to the physis and should inform future studies of human growth spurt.

This study also makes it feasible to directly compare children of both sexes by their timing within their individual growth spurts from hand skeletal radiographic appearance. The growth spurt begins at 85% of final height with all the digits’ metaphyses covered by their respective epiphyses two years before the peak growth. The peak is reached when all the digits are capping at 90% of final height. The growth spurt slows markedly 1.5 years after the peak at 96% of final height when the distal phalanges begin to fuse, and then asymptotically approaches adult height at 3.5 years following the peak growth with closure of the remaining physes.

Because this study was conducted on healthy, middle class, primarily white children, whether this study applicable to current children or is it only of use anthropologically depends on its validity for other populations of children since there is evidence of some maturational differences between the 1930s and today^41^. Repeating this study for modern children is not possible because regular skeletal maturity radiographs can no longer be obtained in longitudinal studies of healthy children because of radiation concerns. However, there are several lines of evidence indicating the data used in this study is highly applicable to modern children. First, we identified sex independent similarities in maturation patterns relative to maturity through the growth spurt. Since sex is a large differentiator in ultimate height growth, identifying these similarities marks a commonality not previously well described. Second, we found nearly identical growth patterns in these cohorts born in the 1920s and 30 s to children born in the 1960s^10^. Third, Paley, *et al*.^19^, in describing the age based multipliers for adult height, utilized data from a number of various databases including the Bolton-Bush and others from various regions including North America, Asia, Africa, Europe and times ranging from the early 1900 s through the 1990 s and found identical age based multipliers in all of the cohorts. Paley’s and our separate analyses provides excellent evidence that even if the absolute values change from place to place or time to time, the relative values of growth remaining are fundamental to human biology and do not appear to change. Finally, comparing the children in these series to the 2000 CDC growth charts shows the children in these series having average heights for all ages above the current 50^th^ percentile.

Ultimately, the utility of this work is in identifying a reliable relationship between skeletal maturation and final adult height as well as being able to standardize where a child is within their individual growth spurt. Because standard growth charts can only evaluate a child’s growth compared to peers, they are not sensitive the amount of growth remaining for any particular child. For example, a child in the 70^th^ percentile in height for age is not expected to reach their adult height faster than a child in the 40^th^ percentile. Other potential data such as secondary sexual characteristics and parents’ heights or age of growth spurt could not be assessed since it was not collected in the original series. The PHV is already considered an important maturity indicator, but it’s timing is difficult to estimate due to diurnal, postural, and measurement variation. Our study clearly identifies the ability to use 90% of adult height as an easily identified, important standard for height growth. With its close relationship to skeletal maturity, the use of 90% adult height as PHV timing surrogate represents a significant advance, as it offers the ability to more reliably quantify maturity and growth remaining in individuals. It also demonstrates the superiority of estimating skeletal maturity using hand radiographs versus chronologic age. Because standard growth charts can only evaluate a child’s growth compared to age matched rather than growth remaining peers, they are not sensitive predictors of remaining growth for any particular child. Because of the wide variability in the age of pubertal onset, a finding of upward or downward crossing of height percentiles in an adolescent is more likely to reflect growth spurt timing rather than a disease state. Clinically, the ability to readily identify a child’s relative height to final maturity should improve both prediction of future growth and identification of pathologic growth delay or acceleration for an individual. This has implications for musculoskeletal care including modulation of spinal or extremity growth, prediction of future limb length difference, and deformity correction via growth modification which all require precise estimates of future growth. Within human biology, biological anthropology, and related fields, the ability to determine future height of a child based on skeletal maturity will likewise be helpful when evaluating skeletal remains.

In summary, we have identified a common pattern of growth between two studies of both boys and girls in geographic diverse areas of the United States in the early 20^th^ century and related them to skeletal maturation of the hand. The timing of the PHV corresponds to 90% of final height, and the stage of the growth spurt corresponds to the hand’s radiological morphology. We have found evidence that this same pattern has existed in other temporally and nationally disparate regions. Despite temporal changes in overall height and age of maturation, we suspect these normalized measures are indicative of healthy children of various times, ethnicity and nationally divergent regions and that skeletal maturity can accurately predict a child’s final height and the amount of growth remaining.

## Electronic supplementary material

Supplemental Information
