# Supplementary material for: The Uniform Pattern of Growth and Skeletal Maturation during the Human Adolescent Growth Spurt
Source: Sci Rep. 2017 Dec 1;7:16705. doi: 10.1038/s41598-017-16996-w (PMC5711808; doi:10.1038/s41598-017-16996-w)
Supplement: Supplementary file 1 — Supplemental Information [file 41598_2017_16996_MOESM1_ESM.doc]

**The Uniform Pattern of Growth and Skeletal Maturation during the Human Adolescent Growth Spurt**

James O. Sanders^1*^, Xing Qiu^2^, Xiang Lu^2^, Dana L. Duren^3^, Raymond W. Liu^4^, Debbie Dang^5^, Mariano E. Menendez^6^, Sarah D. Hans^7^, David R. Weber^8^, Daniel R. Cooperman^9^

^1^Department of Orthopaedics and Rehabilitation, University of Rochester, Rochester, New York

^2^Department of Biostatistics and Computational Biology, University of Rochester, Rochester, New York

^3^Department of Orthopaedic Surgery, University of Missouri, Columbia;

^4^Department of Orthopaedic Surgery, Case Western Reserve University, Cleveland, Ohio

^5^Department of Orthopaedic Surgery, University of California San Francisco;

^6^Department of Orthopedics, Tufts University School of Medicine, Boston, Massachusetts;

^7^Colon Rectal Specialists, Rochester Hills, Michigan;

^8^Department of Pediatrics, University of Rochester, Rochester, New York

^9^Department of Orthopaedics and Rehabilitation, Yale University, New Haven, Connecticut.

*Correspondence to James O. Sanders, MD email: james_sanders@urmc.rochester.edu

| Fels | | | | | | |
| --- | --- | --- | --- | --- | --- | --- |
| Males | | | | Females | | |
| Skeletal Age | % Adult Height | S.D. (%) | Multiplier | % Adult Height | S.D. (%) | Multiplier |
| 7 | 75.01 | 3.349 | 1.333 | 75.95 | 2.985 | 1.317 |
| 7.5 | 75.27 | 3.225 | 1.328 | 77.28 | 2.907 | 1.294 |
| 8 | 75.79 | 2.975 | 1.319 | 78.54 | 2.853 | 1.273 |
| 8.5 | 76.64 | 2.73 | 1.305 | 79.74 | 2.784 | 1.254 |
| 9 | 77.71 | 2.567 | 1.287 | 80.93 | 2.643 | 1.236 |
| 9.5 | 78.87 | 2.529 | 1.268 | 82.16 | 2.416 | 1.217 |
| 10 | 80.00 | 2.548 | 1.250 | 83.49 | 2.193 | 1.198 |
| 10.5 | 81.09 | 2.553 | 1.233 | 84.97 | 2.069 | 1.177 |
| 11 | 82.22 | 2.495 | 1.216 | 86.66 | 2.061 | 1.154 |
| 11.5 | 83.47 | 2.357 | 1.198 | 88.62 | 2.119 | 1.128 |
| 12 | 84.85 | 2.205 | 1.178 | 90.88 | 2.107 | 1.100 |
| 12.5 | 86.39 | 2.104 | 1.158 | 93.32 | 1.956 | 1.072 |
| 13 | 88.16 | 2.059 | 1.134 | 95.47 | 1.742 | 1.047 |
| 13.5 | 90.20 | 2.051 | 1.109 | 97.02 | 1.541 | 1.031 |
| 14 | 92.27 | 2.023 | 1.084 | 98.06 | 1.376 | 1.020 |
| 14.5 | 94.17 | 1.932 | 1.062 | 98.72 | 1.244 | 1.013 |
| 15 | 95.77 | 1.773 | 1.044 | 99.16 | 1.08 | 1.008 |
| 15.5 | 97.02 | 1.555 | 1.031 | 99.48 | 0.839 | 1.005 |
| 16 | 97.93 | 1.332 | 1.021 | 99.69 | 0.601 | 1.003 |
| 16.5 | 98.56 | 1.155 | 1.015 | 99.78 | 0.489 | 1.002 |
| 17 | 99.02 | 0.988 | 1.010 | 99.83 | 0.447 | 1.002 |
| 17.5 | 99.36 | 0.791 | 1.006 | 99.86 | 0.388 | 1.001 |
| 18 | 99.56 | 0.637 | 1.004 | 99.88 | 0.351 | 1.001 |

Supplemental Table 1 Fels Skeletal ages compared to percentage final heights and multipliers

| Greulich and Pyle - Males | | | |
| --- | --- | --- | --- |
| Skeletal Age | % Adult Height | S.D. (%) | Multiplier |
| 7yr | 73.30 | 2.026 | 1.364 |
| 8yr | 72.08 | 2.543 | 1.387 |
| 9yr | 77.16 | 2.66 | 1.296 |
| 10yr | 80.07 | 2.529 | 1.249 |
| 11yr | 82.59 | 2.514 | 1.211 |
| 11yr 6mo | 84.08 | 2.729 | 1.189 |
| 12yr 6mo | 87.99 | 2.934 | 1.136 |
| 13yr | 90.49 | 2.699 | 1.105 |
| 13yr 6mo | 93.14 | 2.168 | 1.074 |
| 14yr | 95.39 | 1.488 | 1.048 |
| 15yr | 97.69 | 0.88 | 1.024 |
| 15yr 6mo | 98.58 | 0.768 | 1.014 |
| 16yr | 99.28 | 0.642 | 1.007 |
| 17yr | 99.70 | 0.441 | 1.003 |
| 18yr | 99.87 | 0.233 | 1.001 |
| 19yr | 99.81 | 0.237 | 1.002 |

Supplemental Table 2 Greulich and Pyle male skeletal ages compared to percentage final heights and multipliers

| Greulich and Pyle - Females | | | |
| --- | --- | --- | --- |
| Skeletal Age | % Adult Height | S.D. (%) | Multiplier |
| 4yr 2mo | 66.27 | 0.256 | 1.509 |
| 5yr | 69.75 | 1.424 | 1.434 |
| 5yr 9mo | 69.97 | 2.708 | 1.429 |
| 6yr 10mo | 73.63 | 2.512 | 1.358 |
| 7yr 10mo | 76.56 | 2.768 | 1.306 |
| 8yr 10mo | 80.80 | 2.146 | 1.238 |
| 10yr | 84.79 | 2.003 | 1.179 |
| 11yr | 88.43 | 2.901 | 1.131 |
| 12yr | 91.34 | 2.7 | 1.095 |
| 13yr | 95.10 | 1.902 | 1.052 |
| 13yr 6mo | 97.09 | 1.421 | 1.030 |
| 14yr | 98.58 | 1.005 | 1.014 |
| 15yr | 99.33 | 1.133 | 1.007 |
| 16yr | 99.93 | 0.465 | 1.001 |
| 17yr | 99.73 | 0.297 | 1.003 |
| 18yr | 99.91 | 0.06 | 1.001 |

Supplemental Table 3 Greulich and Pyle female skeletal ages compared to percentage final heights and multipliers

| Sanders | | | | | | |
| --- | --- | --- | --- | --- | --- | --- |
| Males | | | | Females | | |
| Skeletal Stage | % Adult Height | S.D. (%) | Multiplier | % Adult Height | S.D. (%) | Multiplier |
| 2 | 87.21 | 2.535 | 1.147 | 86.15 | 2.001 | 1.161 |
| 3A | 91.56 | 1.429 | 1.092 | 90.93 | 2.276 | 1.100 |
| 3B | 94.21 | 2.05 | 1.062 | 92.53 | 2.315 | 1.081 |
| 4 | 97.20 | 0.876 | 1.029 | 96.19 | 0.906 | 1.040 |
| 5 | 98.27 | 0.531 | 1.018 | 97.21 | 0.899 | 1.029 |
| 6 | 98.58 | 0.758 | 1.014 | 98.51 | 0.766 | 1.015 |
| 7 | 99.61 | 0.493 | 1.004 | 99.46 | 1.064 | 1.005 |
| 8 | 99.85 | 0.218 | 1.002 | 99.85 | 0.269 | 1.001 |

Supplemental Table 4 Sanders skeletal maturity stages compared to percentage final heights and multipliers

Supplemental Figure 1. Percent Adult Height compared to Fels Skeletal Ages. The red bars represent standard deviations. Supplemental Figure 1A is males and 1B females.


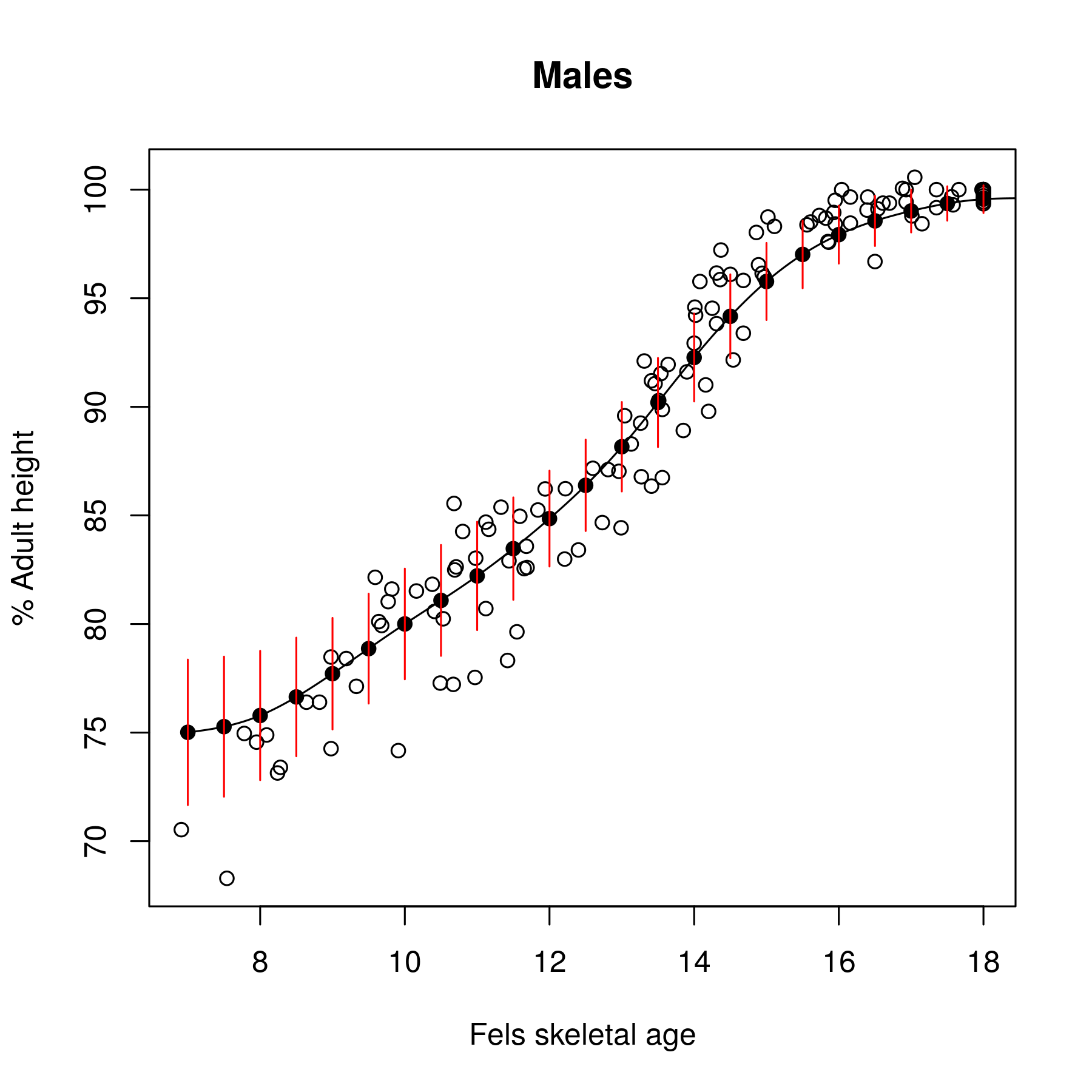


Supplemental Figure 1A: Males


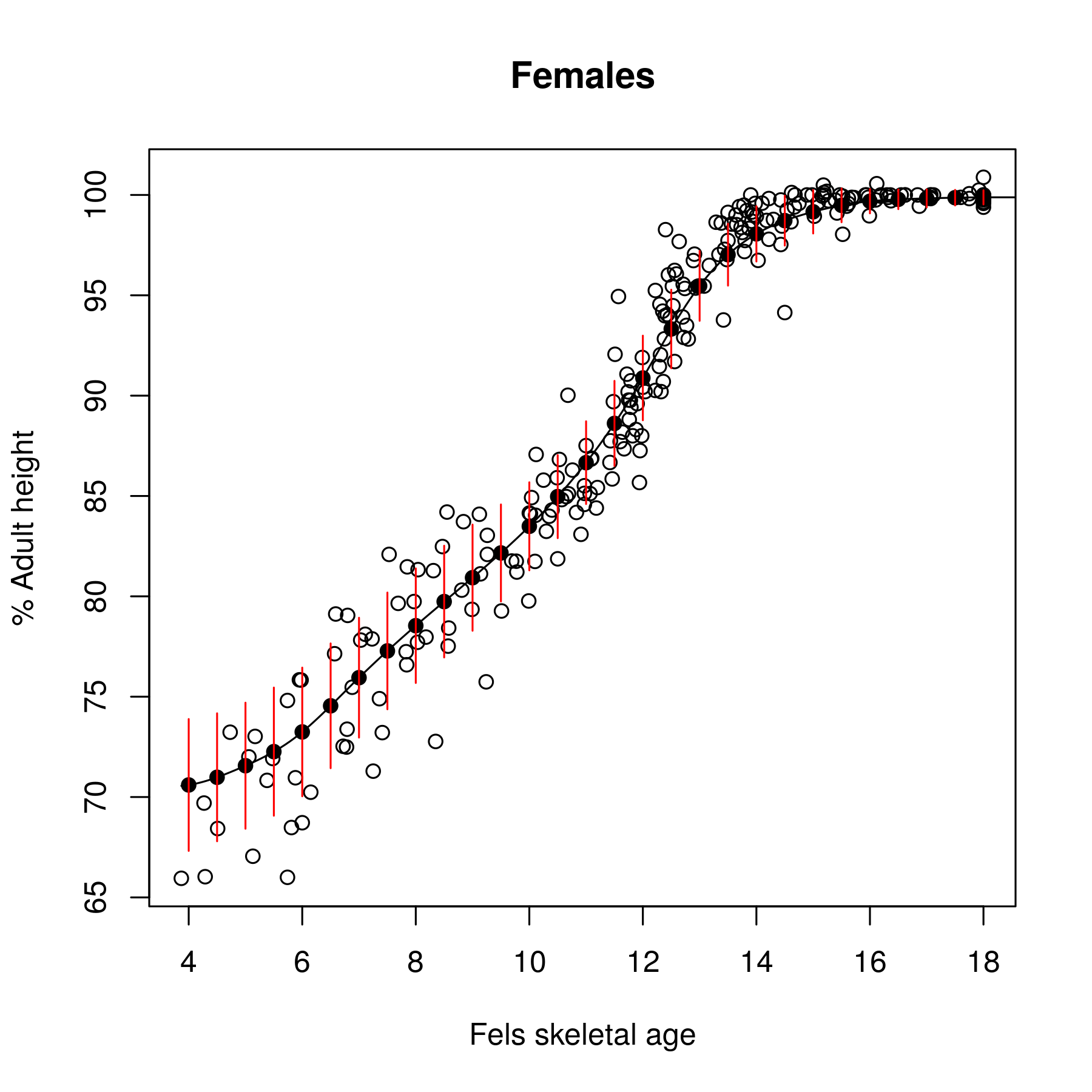


Supplemental Figure 1B: Females

Supplemental Figure 2. Percent Adult Height compared to Greulich and Pyle Skeletal Ages. The red bars represent standard deviations. Supplemental Figure 2A is males and 2B females.


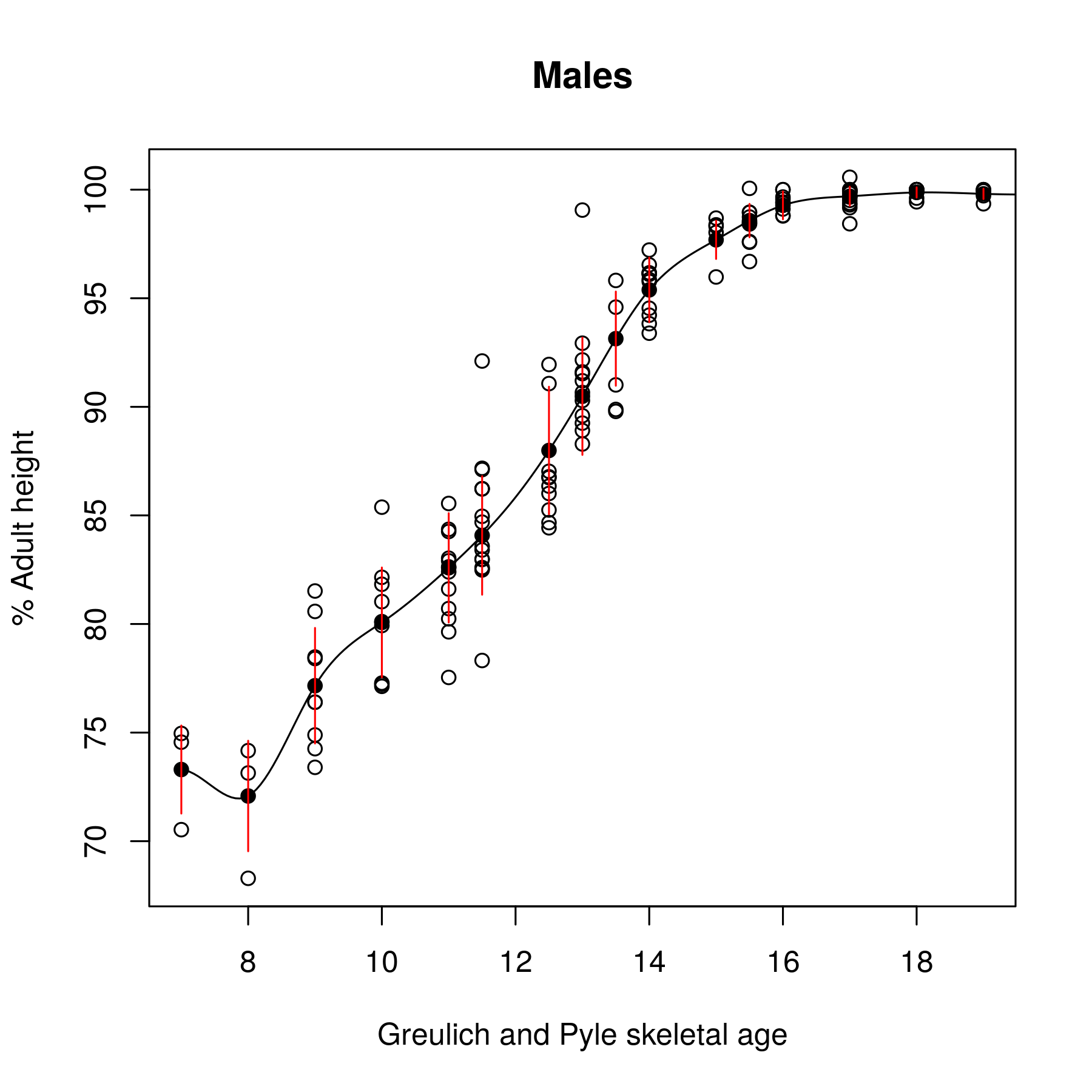


Supplemental Figure 2A: Males


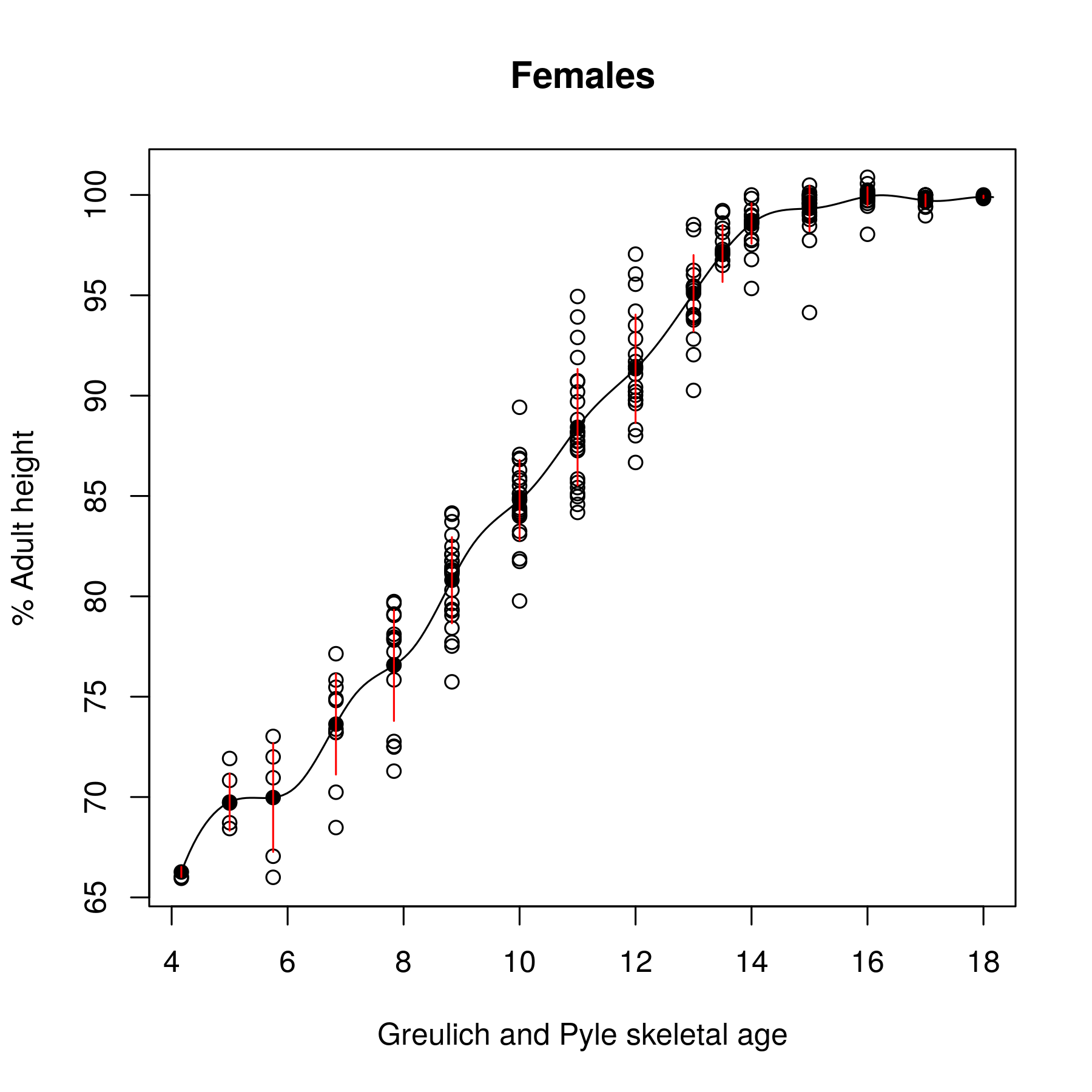


Supplemental Figure 2B: Females

Supplemental Figure 3. Percent Adult Height compared to Sanders skeletal stages. There is a clear floor effect below stage 2. The red bars represent standard deviations. Supplemental Figure 3A is males and 3B females.


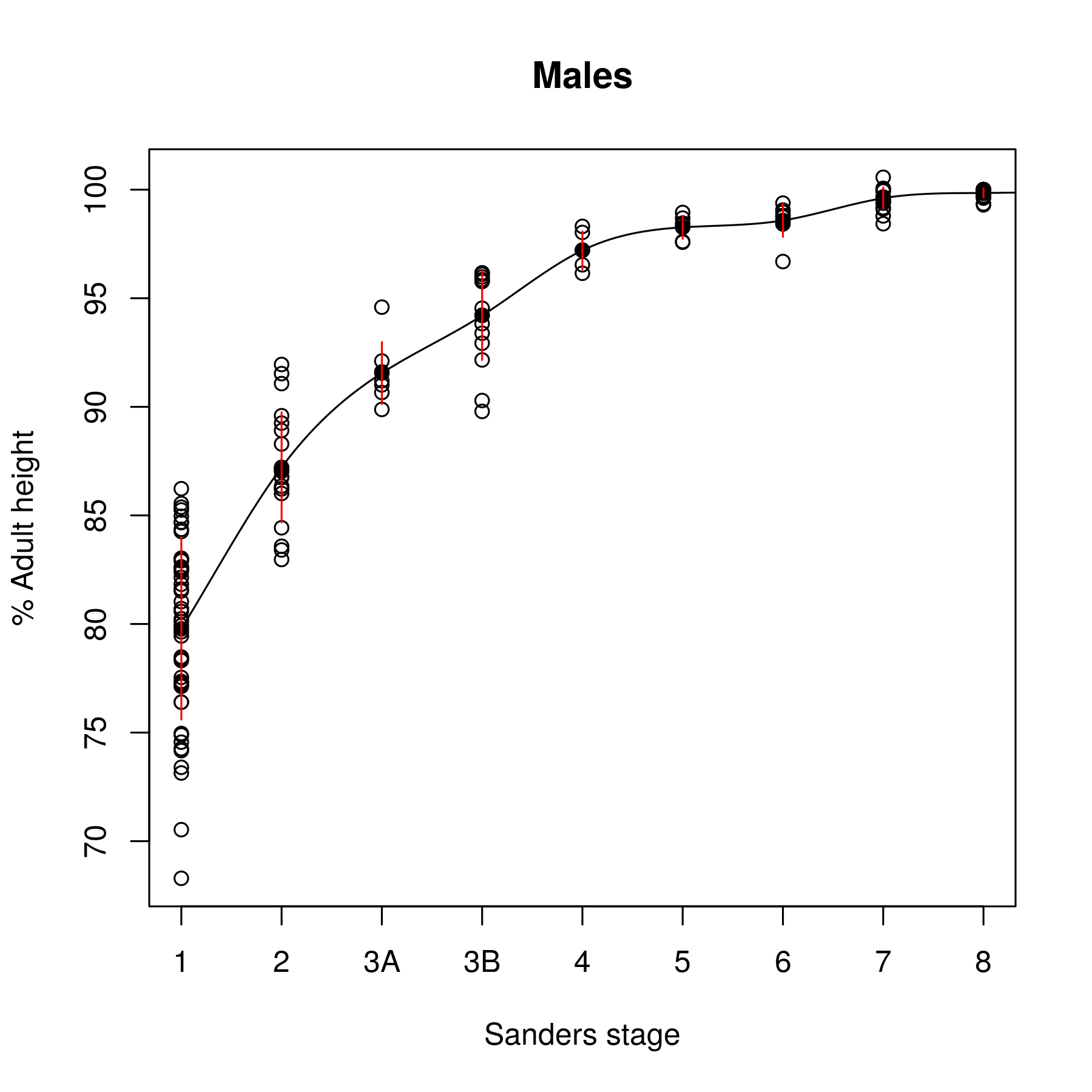


Supplemental Figure 3A: Males


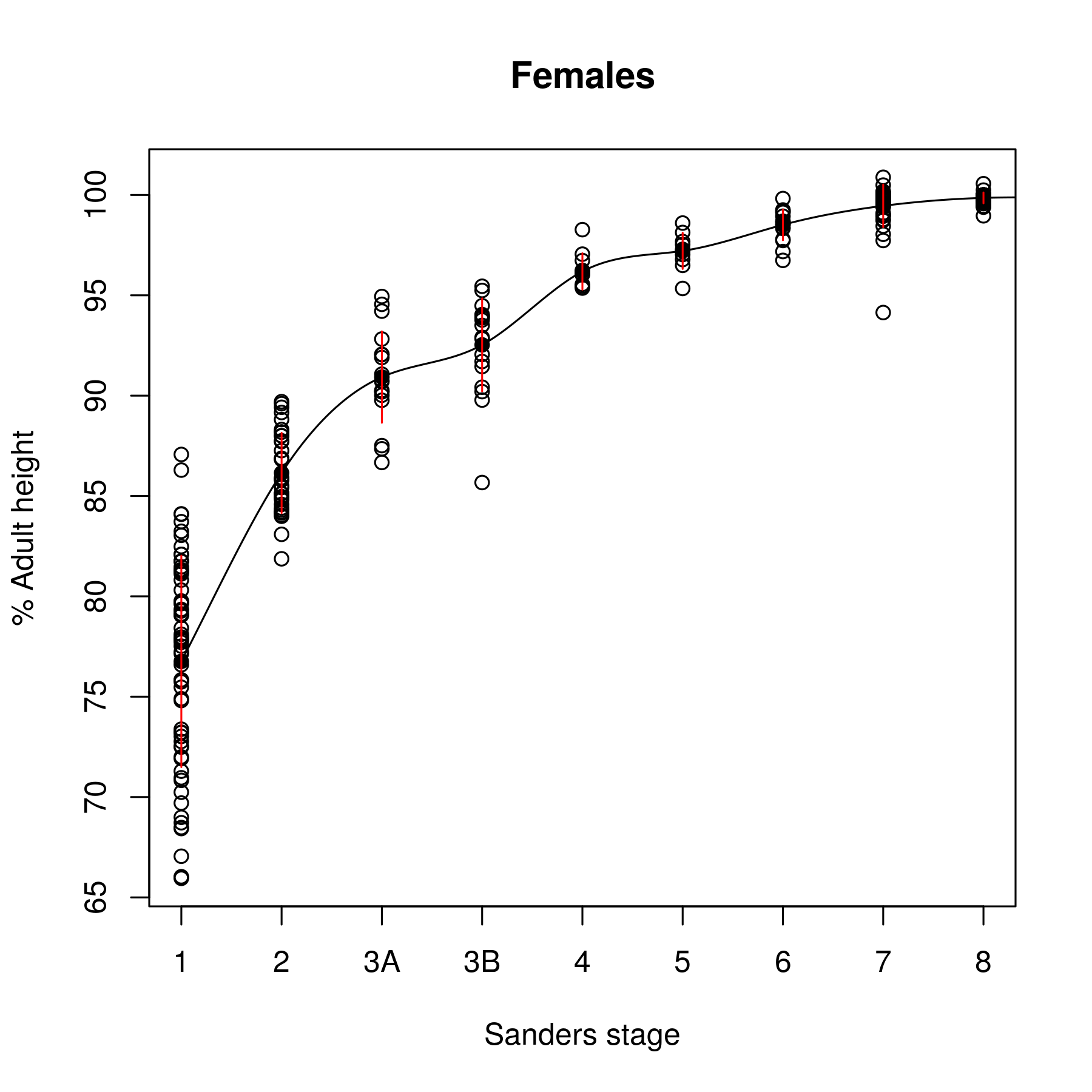


Supplemental Figure 3B: Females

Supplemental Figure 4. Timing in years relative to the peak growth compared to Fels skeletal age. The red bars represent standard deviations. Supplemental Figure 4A is males and 4B females.


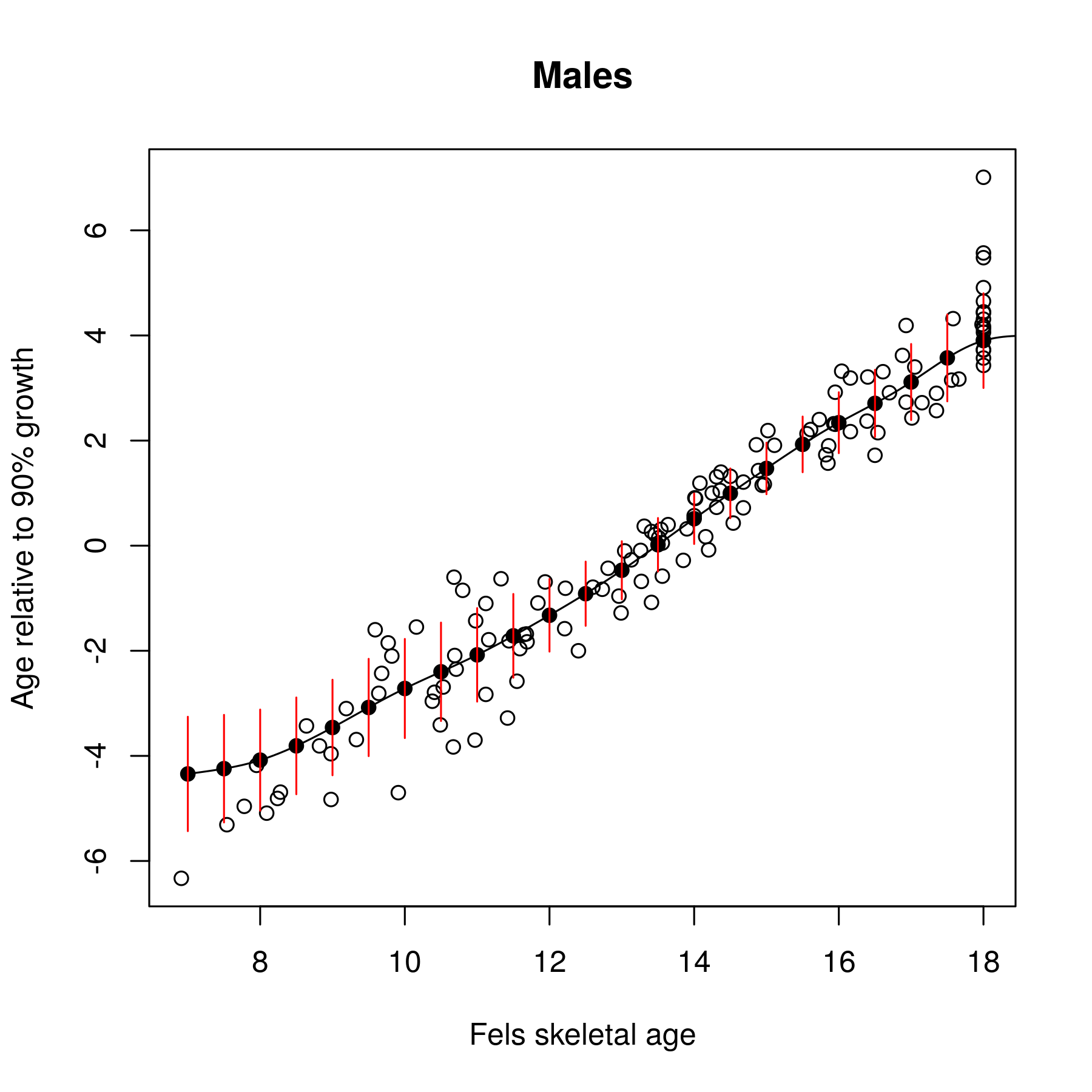


Supplemental Figure 4A: Males


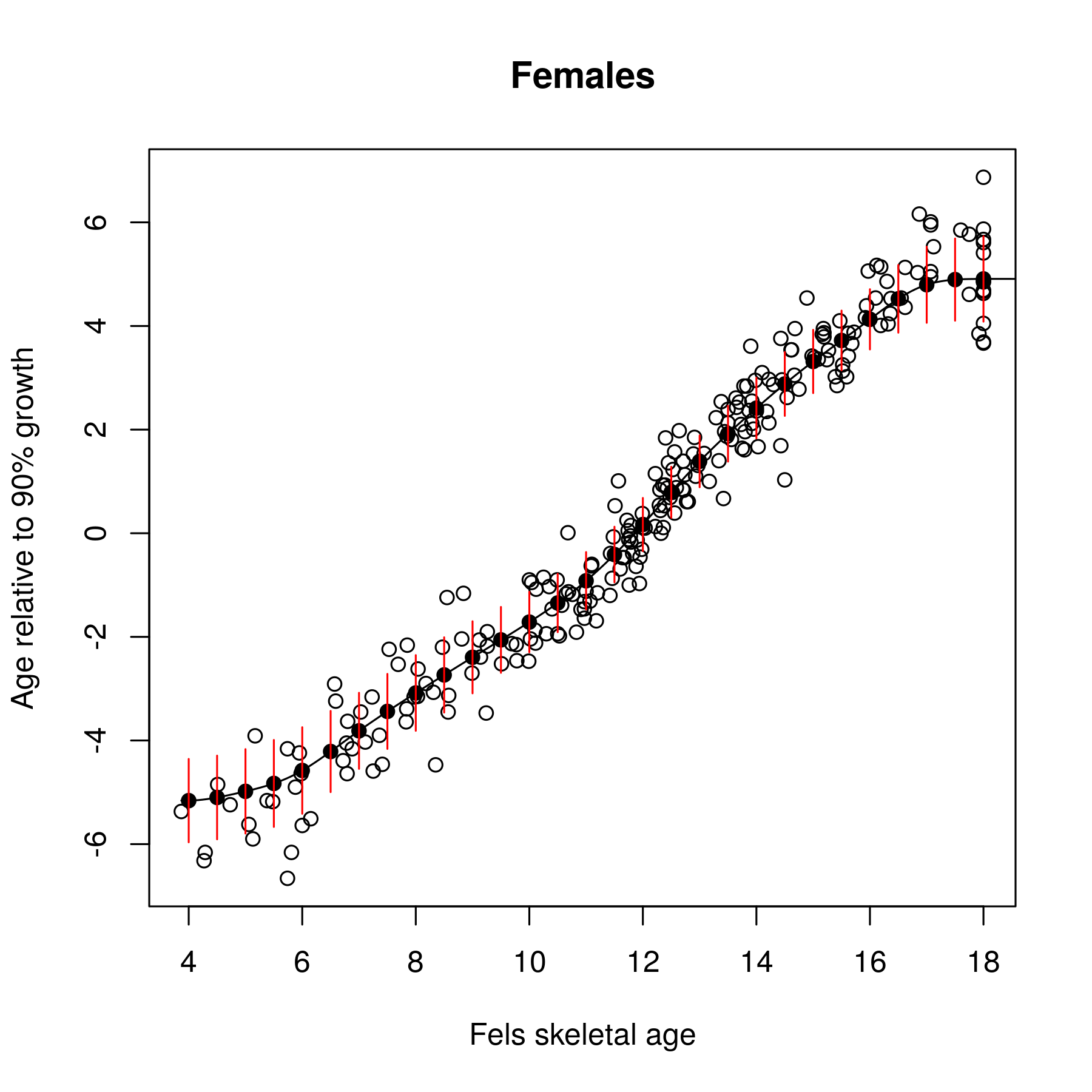


Supplemental Figure 4B: Females

Supplemental Figure 5. Timing in years relative to the peak growth compared to Greulich and Pyle skeletal age. The red bars represent standard deviations. Supplemental Figure 5A is males and 5B females


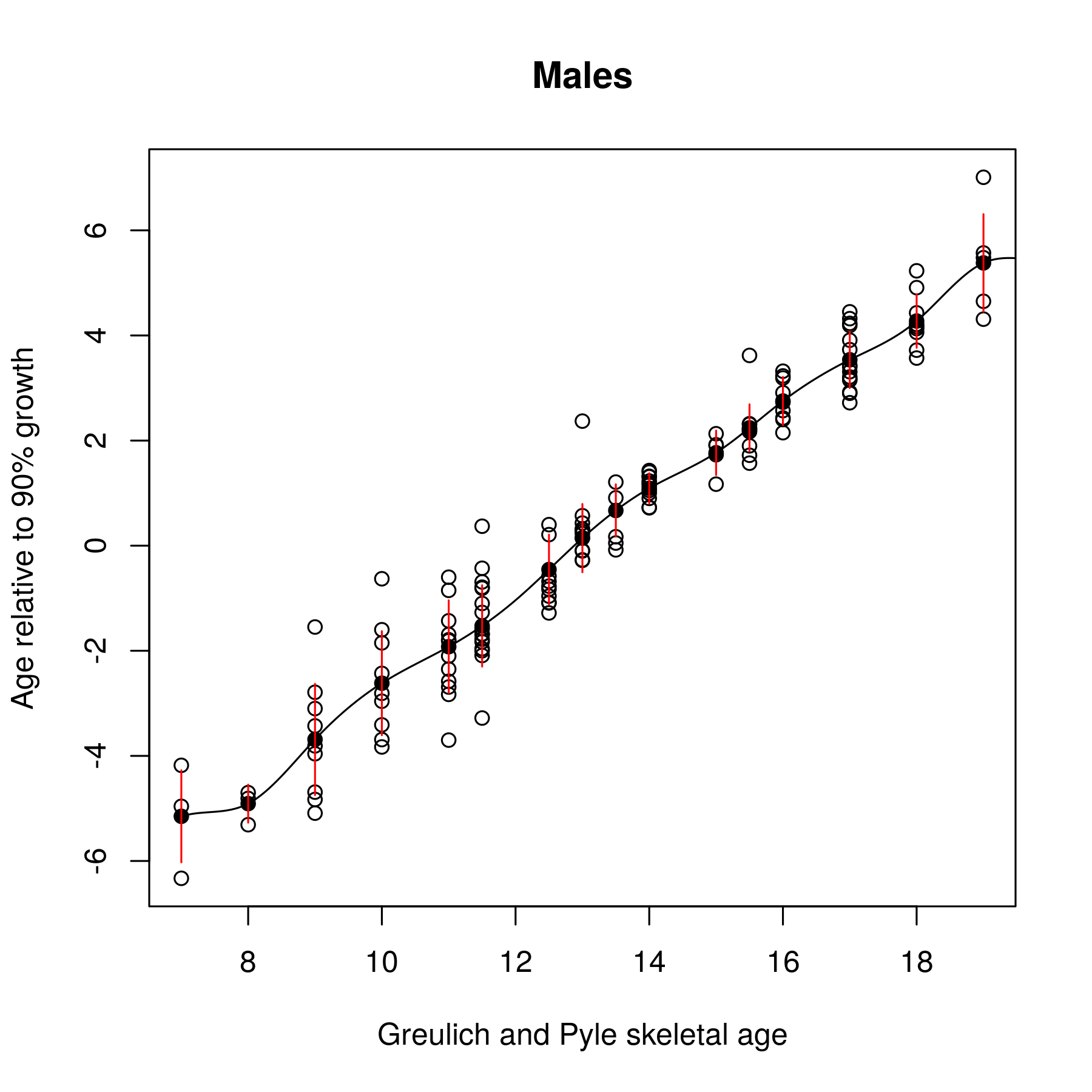


Supplemental Figure 5A: Males


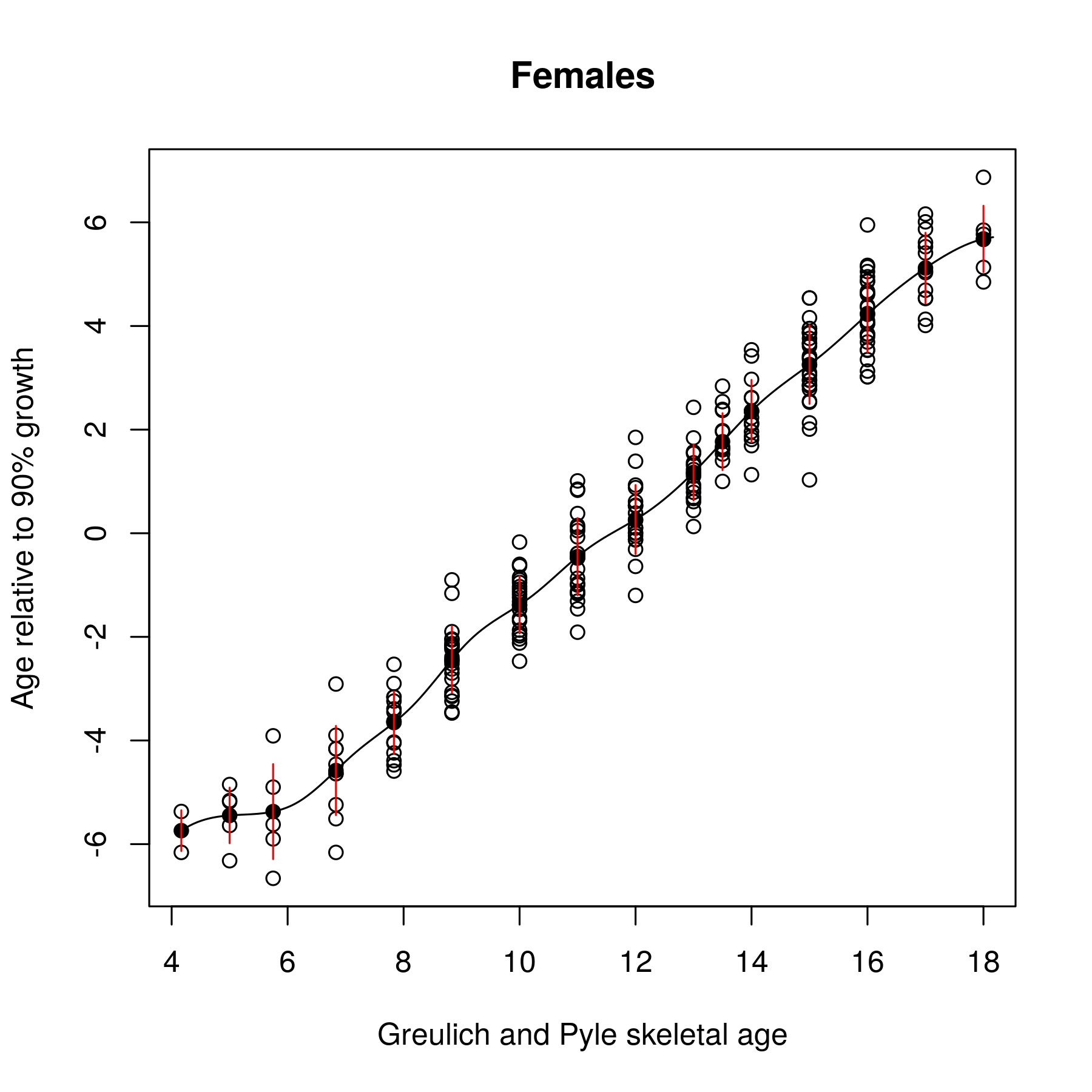


Supplemental Figure 5B: Females

Supplemental Figure 6 Timing in years relative to the peak growth compared to Sanders skeletal stage. There is a clear floor effect below stage 2. The red bars represent standard deviations. Supplemental Figure 6A is males and 6B females


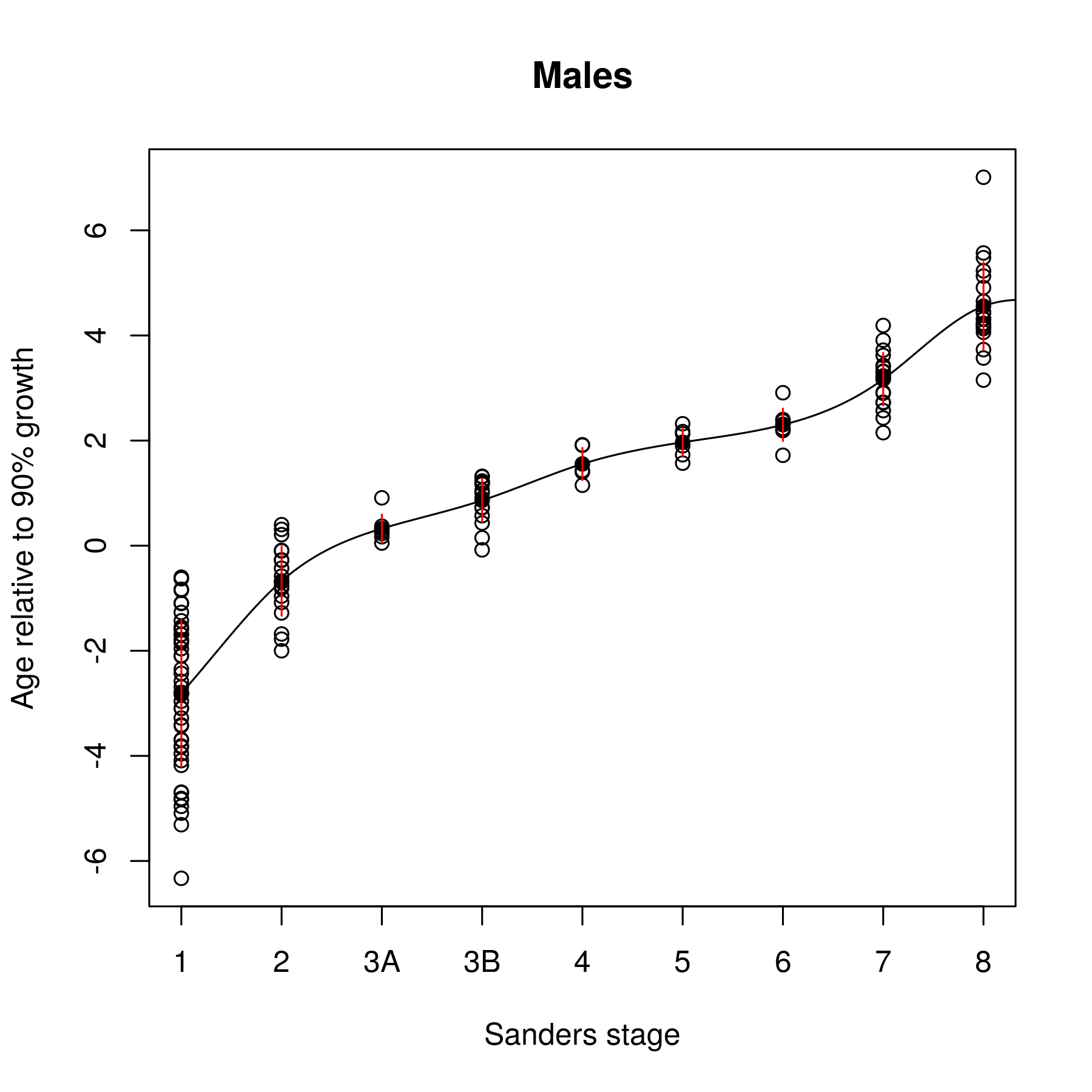


Supplemental Figure 6A: Males


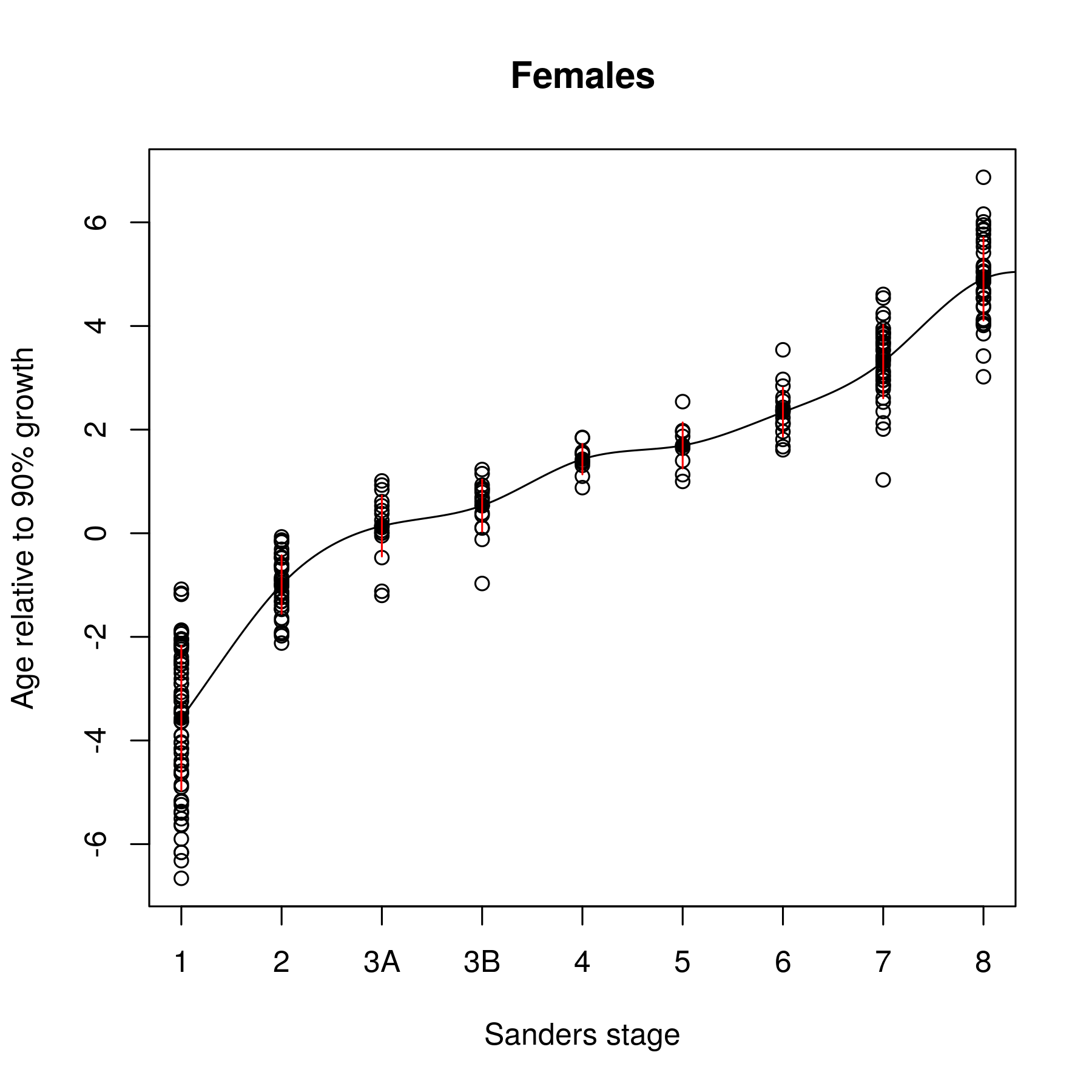


Supplemental Figure 6B Females
